# Role of the kallikrein–kinin system in traumatic brain injury

**DOI:** 10.3389/fncel.2014.00345

**Published:** 2014-11-03

**Authors:** Christiane Albert-Weissenberger, Stine Mencl, Sarah Hopp, Christoph Kleinschnitz, Anna-Leena Sirén

**Affiliations:** ^1^Department of Neurology, University Hospital of WürzburgWürzburg, Germany; ^2^Department of Neurosurgery, University Hospital of WürzburgWürzburg, Germany

**Keywords:** traumatic brain injury, kallikrein–kinin system, bradykinin, factor XII, kinin receptor

## Abstract

Traumatic brain injury (TBI) is a major cause of mortality and morbidity worldwide. Despite improvements in acute intensive care, there are currently no specific therapies to ameliorate the effects of TBI. Successful therapeutic strategies for TBI should target multiple pathophysiologic mechanisms that occur at different stages of brain injury. The kallikrein–kinin system is a promising therapeutic target for TBI as it mediates key pathologic events of traumatic brain damage, such as edema formation, inflammation, and thrombosis. Selective and specific kinin receptor antagonists and inhibitors of plasma kallikrein and coagulation factor XII have been developed, and have already shown therapeutic efficacy in animal models of stroke and TBI. However, conflicting preclinical evaluation, as well as limited and inconclusive data from clinical trials in TBI, suggests that caution should be taken before transferring observations made in animals to humans. This review summarizes current evidence on the pathologic significance of the kallikrein–kinin system during TBI in animal models and, where available, the experimental findings are compared with human data.

## Introduction

Traumatic brain injury (TBI) accounts for one-third of all injury-related deaths. An estimated 1.74 million TBIs occur annually in the United States (Faul et al., 2010; Ma et al., 2014). About 43% of people discharged with TBI after acute hospitalization, develop TBI-related long-term disability. Moreover, individuals with a history of TBI are more likely to receive welfare or disability payments and to develop neurologic disorders that are disabling in their own right (Ma et al., 2014)— for example, Alzheimer’s disease (Fleminger et al., 2003). The incidence of TBI is particularly high in younger age groups, with motor vehicle accidents being the leading cause (Asemota et al., 2013). The direct costs of TBI have been estimated at $13.1 billion per year (in 2013) in the United States (Ma et al., 2014); additionally, $64.7 billion per year are lost through missed work and lost productivity, and total medical costs range from $63.4 to $79.1 billion per year (Ma et al., 2014). The significant economic impact of TBI is at variance with the lack of therapies available to ameliorate the effects of TBI.

To better understand the pathobiology of TBI and to evaluate potential therapeutic approaches, various animal models have been developed to mimic certain components of clinical TBI. Closed-head weight-drop models—with a weight that falls onto the exposed skull—probably mimic most closely clinical TBI cases. Depending on the experimental settings, the impact of the weight results in largely focal or diffuse brain injury. In controlled cortical impact models an impact onto the dura, inflicted by a pneumatic pistol, predominantly results in focal brain injury. For fluid percussion models it is inconsistently reported to what extend the brain injury is diffuse or focal. Here, tissue damage is induced by a fluid pulse onto the intact dura through a craniotomy. A solely focal brain injury can be achieved by cold lesion models, which commonly utilize a cold rod that is exposed to the dura or skull (for a comprehensive review, see Albert-Weissenberger and Sirén, 2010). Despite promising results from these experimental TBI models, more than 30 phase III trials of TBI in humans have failed to generate favorable results in terms of developing potential therapeutic strategies (Doppenberg et al., 2004; Maas et al., 2010). In part, these failures likely reflect the heterogeneity of TBI (e.g., severity and location of the injury—focal vs. diffuse injury). Therefore, future therapeutic approaches are more likely to succeed if they target diverse pathophysiologic mechanisms. As the kallikrein–kinin system links edema formation, inflammation, and thrombosis (Costa-Neto et al., 2008; Langhauser et al., 2012), it seems to be a promising target.

In this review, current available evidence on the pathologic significance of the kallikrein–kinin system during TBI is summarized. Findings from experimental models are compared with human data, where available.

## The kallikrein–kinin system

**Figure 1:**
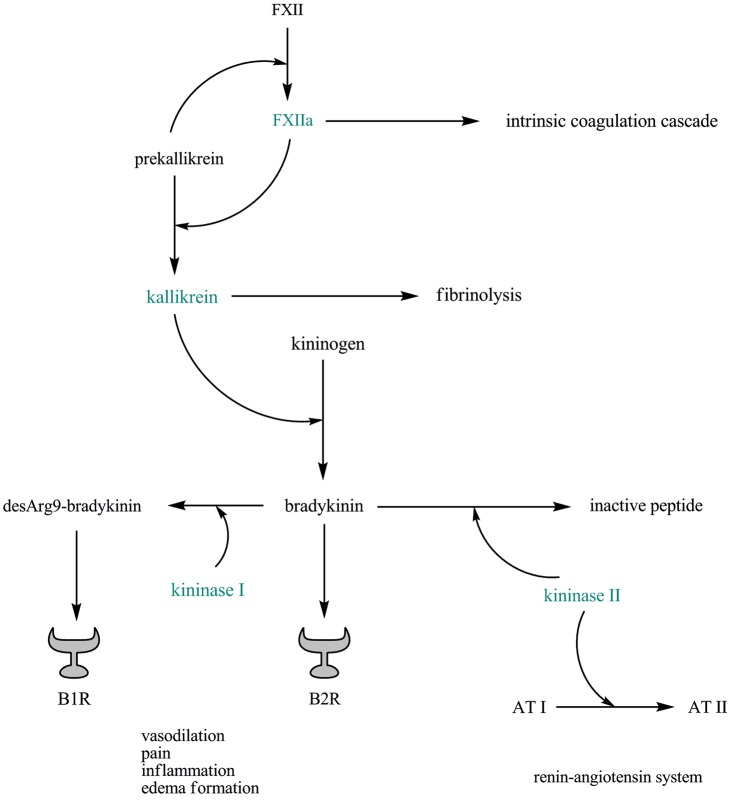
**The plasma kallikrein–kinin system is linked to thrombosis, fibrinolysis, and the renin–angiotensin system**. Abbreviations: AT, angiotensin; B1R, kinin receptor B1; B2R, kinin receptor B2; FXII, factor XII; FXIIa, activated factor XII.

Kinins play key roles in regulating vascular permeability and inflammatory processes following tissue injury (Leeb-Lundberg et al., 2005). They are released either by the tissue or the plasma. In the tissue, kallikrein is activated by proteases and it releases a kinin called kallidin from the inactive precursors, the kininogens. Plasma kallikrein is released from prekallikrein by activated factor XII (FXII) and reciprocally activates FXII (Revak et al., 1978). Subsequently, plasma kallikrein releases bradykinin from the kininogens. Kallidin and bradykinin mediate their effects via kinin receptor B2. Both kallidin and bradykinin are converted by the action of kininase I-type carboxypeptidases into des-Arg9-bradykinin and des-Arg10-kallidin, respectively, which specifically bind to kinin receptor B1 (Figure 1).

Interestingly, the plasma kallikrein–kinin system is linked to thrombosis, fibrinolysis, and the renin–angiotensin system: FXII has an essential role in thrombosis (Renné et al., 2012), and mice selectively depleted of plasma kallikrein or FXII are protected from pathogenic thrombus formation without increased risk of bleeding (Revenko et al., 2011). Plasma kallikrein (and, to a lesser extent, activated FXII) converts plasminogen to plasmin, linking the kallikrein–kinin system to fibrinolysis (Colman, 1969). In addition, bradykinin is mainly inactivated by kininase II (also known as angiotensin converting enzyme (ACE)), an enzyme that also degrades angiotensin I into angiotensin II (Bernstein et al., 2011; Figure 1).

## Role of the kinin receptors in traumatic brain injury

All essential components of the kallikrein–kinin system are present in the rodent and human brain (Kariya et al., 1985; Kizuki et al., 1994; Ongali et al., 2003; Trabold et al., 2010). Moreover, it has been reported that their expression is induced after brain injury but the expression pattern varies depending on the brain injury model used (Ongali et al., 2006; Raslan et al., 2010; Trabold et al., 2010; Albert-Weissenberger et al., 2012). In a controlled cortical impact model, bradykinin concentrations in the brain were significantly increased at 2 h post-injury, and then subsequently declined (Trabold et al., 2010). Kinin receptor B1 transcripts peaked at 6 h post-injury and remained elevated until day 2, whereas kinin receptor B2 was constitutively expressed at lower levels (Trabold et al., 2010). In a cold lesion model, a strong but transient mRNA expression of kinin receptor B1 was observed in the first 12 h after injury, whereas the enhanced mRNA expression of kinin receptor B2 was more sustained, lasting up to 48 h (Raslan et al., 2010). In our hand, a closed-head weight-drop trauma in mice resulting in a mixed brain injury pattern (focal and diffuse brain injury) caused a slight increase of kinin receptor mRNA levels one week after injury induction (Albert-Weissenberger et al., 2012).

Kinins mediate their physiologic effects via kinin receptors B1 and B2. Support for a pathologic role of kinin receptors in TBI was obtained through the use of genetically engineered mice that lack either kinin receptor B1 or kinin receptor B2. After controlled cortical impact, kinin receptor B2-deficient mice, but not kinin receptor B1-deficient mice, had less brain edema, smaller lesion volumes, and a better functional outcome as compared with wild-type mice (Trabold et al., 2010). Another study also reported that the kinin receptor B2 mediates detrimental effects after TBI in mice (Hellal et al., 2003). On the contrary, findings from our group point out that kinin receptor B1 plays an important role in the pathophysiology of TBI (Raslan et al., 2010). Kinin receptor B1-deficient mice subjected to cold lesion displayed smaller lesion volumes, less blood–brain barrier disruption, and less inflammation in the injured brain area, whereas kinin receptor B2-deficient mice were fully susceptible to brain trauma. Supporting these results, application of the kinin receptor B1-inhibitor R-715 reduced lesion size even in a therapeutic setting (administered 1 h after injury induction), whereas application of the kinin receptor B2-inhibitor Hoe140 (Icatibant) had no significant effect on lesion volume in wild-type mice (Raslan et al., 2010). Importantly, application of the kinin receptor B2-inhibitor Hoe140 in kinin receptor B1-deficient mice had no additive benefit on the reduction in brain lesion size. However, Hoe140 treatment has been shown to result in a moderate reduction in brain lesion size after cold lesion in rats and mice (by 19% and 14%, respectively) (Görlach et al., 2001). We recently reported that kinin receptor B1 deficiency in mice is associated with diminished functional deficits and a reduction in axonal injury, astrogliosis, and neuronal apoptosis after a weight-drop-induced brain trauma (Albert-Weissenberger et al., 2012). Inhibition of kinin receptor B1 in wild-type mice by the specific kinin receptor B1-blocker R-715, starting from 1 h after trauma, confirmed these results. By contrast, deficiency of kinin receptor B2 was ineffective in this trauma model (Albert-Weissenberger et al., 2012).

Kinin receptor inhibitors, other than the kinin receptor B1-inhibitor R-715 and the kinin receptor B2-inhibitor Hoe140, have also been tested in experimental and clinical settings of TBI. Treatment with the kinin receptor B2-antagonist LF 18-1505T resulted in reduced brain edema and improved neurologic outcome in a closed-head trauma model in rats (Ivashkova et al., 2006). In rats subjected to closed-head trauma, a continuous infusion of the nonpeptide kinin receptor B2-inhibitor LF 16-0687 (Anatibant), from 1 h to 24 h after injury, resulted in diminished brain edema formation on day 1 and less neurologic deficits on day 1, day 3, and day 7 (Pruneau et al., 1999). Administered as a single dose 1 h after trauma, LF 16-0687 was able to reduce brain swelling and to improve the recovery of neurologic function following closed-head trauma in rats (Kaplanski et al., 2002). Similar results were obtained after controlled cortical impact or cold lesion (Schulz et al., 2000; Stover et al., 2000; Zweckberger and Plesnila, 2009). It was suggested that stabilization of the blood–brain barrier and mitigation of inflammatory processes are the underlying mechanisms. However, it remains questionable whether LF 16-0687 is effective within a clinically relevant time window (Plesnila et al., 2001). LF 16-0687 was investigated in a phase I clinical study (Marmarou et al., 2005) in patients with severe TBI. In this trial, patients with TBI and Glasgow Coma Scale <8 received LF 16-0687 as a single subcutaneous injection within 8–12 h after TBI, and Marmarou et al. concluded that LF 16-0687 provides a potential therapeutic approach to treating cerebral edema following brain damage, as the compound was well tolerated. A phase II trial using LF 16-0687 in TBI patients with a Glasgow Coma Scale score of ≤12 was unable to recruit a sufficient number of patients (Shakur et al., 2009). Moreover, results from this trial were disappointing in that there was a non-significant trend towards worse outcomes in the LF 16-0687 treatment group.

Bradycor (Deltibant, CP-0127), a peptide compound kinin receptor B2-antagonist, was tested in a pilot, single-blinded clinical pilot study in 20 patients with focal head injury. Results indicated that CP-0127 treatment diminished the pathologic rise of intracranial pressure (Narotam et al., 1998). A phase II trial in severely brain injured patients reported a slight trend towards a better outcome in the CP-0127 treatment group (Marmarou et al., 1999). However, a Cochrane analysis concluded that those clinical trials do not provide reliable evidence that kinin receptor B2-antagonists are effective in improving outcome after TBI (Ker and Blackhall, 2008). Reports on the clinical use of kinin receptor B1-inhibitors in patients with TBI are not yet available.

## Role of the kallikreins and factor XII in traumatic brain injury

In 1978, it was reported that patients with severe trauma have increased protease activity in the cerebrospinal fluid, the activity of which could be inhibited by aprotinin treatment. In rabbits subjected to cold injury, aprotinin treatment resulted in reduced brain edema formation (Unterberg et al., 1986). Aprotinin is known to inhibit several serine proteases, including plasma kallikrein, and a reduced protease activity has been associated with a lower mortality rate (Auer et al., 1979).

There are promising results from recent studies suggesting a therapeutic potential for the serine protease inhibitor C1-inhibitor. C1-inhibitor is an endogenous regulator with various physiologic functions (Singer and Jones, 2011), including the inhibition of activated FXII and plasma kallikrein. Application of C1-inhibitor has proven to be beneficial in ischemic stroke (Heydenreich et al., 2012). Similarly, in mice subjected to controlled cortical impact, C1-inhibitor treatment at 10 min (Longhi et al., 2008) or 1 h (Longhi et al., 2009) after injury resulted in less pronounced functional deficits and smaller brain lesions compared with control mice.

## Role of the kininase II in traumatic brain injury

Indirect support for a pathologic role of kinins in TBI was obtained through inhibition of kininase II, an enzyme that hydrolyzes proteins such as bradykinin, substance P, and angiotensin I. Inhibition of kininase II results in downregulation of angiotensin II production. Moreover, it has been reported that kininase II inhibition potentiates the physiologic effects of kinins and all kinin-related peptides are subject to less hydrolyzation. Using the kininase II inhibitor Captopril, Harford-Wright et al. (2010) showed, in a diffuse TBI model, that inhibition of kininase II results in increased “dark cell changes” and in exacerbated motor function deficits. However, they did not consider the effects of kininase II inhibition on the kallikrein–kinin system; instead, the authors conclude that kininase II inhibitors worsen outcome following TBI, presumably because they impair the degradation of substance P—as shown by the increase in substance P immunoreactivity. However, the fact that bradykinin potentiates the release of substance P should also be noted (for a review, see Geppetti, 1993). Interestingly, a kininase II polymorphism in humans influences the neuropsychologic subacute performance of patients with moderate or severe TBI (Ariza et al., 2006).

## Perspective

There is accumulating evidence that the kallikrein–kinin system is critically involved in various brain diseases (e.g., stroke, multiple sclerosis, Alzheimer’s disease, epilepsy, depression) and its modulation might be a promising strategy to combat these diseases. The reported effects of specific components of the kallikrein–kinin system, however, are often inconsistent.

The paucity of therapies for brain trauma has resulted in a pressing clinical demand for new treatment options. The findings summarized in this review indicate that modulation of the components of the kallikrein–kinin system, which links edema formation, inflammation, and thrombosis, might be a promising strategy to combat TBI. Another tempting approach might be inhibition of the starting point of the kallikrein–kinin system, e.g., by the C1-inhibitor.

## Author contributions

Christiane Albert-Weissenberger wrote the manuscript. Stine Mencl corrected the manuscript and checked the references. Sarah Hopp corrected the manuscript and build the figure. Christoph Kleinschnitz revised the manuscript for important intellectual content and approved the final version of the manuscript. Anna-Leena Sirén revised the manuscript critically for important intellectual content and approved the final version of the manuscript.

## Conflict of interest statement

The Review Editor Dr. Tim Magnus declares that, despite having collaborated with the author Dr. Christoph Kleinschnitz, the review process was handled objectively and no conflict of interest exists. The authors declare that the research was conducted in the absence of any commercial or financial relationships that could be construed as a potential conflict of interest.
